# *Theileria equi* claudin like apicomplexan microneme protein contains neutralization-sensitive epitopes and interacts with components of the equine erythrocyte membrane skeleton

**DOI:** 10.1038/s41598-021-88902-4

**Published:** 2021-04-29

**Authors:** Cynthia K. Onzere, Lindsay M. Fry, Richard P. Bishop, Marta G. Silva, Reginaldo G. Bastos, Donald P. Knowles, Carlos E. Suarez

**Affiliations:** 1 Department of Veterinary Microbiology and Pathology, Washington State University, P.O. Box 647040, Pullman, WA 99164 USA; 2 Animal Disease Research Unit, Agricultural Research Service, US Department of Agriculture, P.O. Box 646630, Pullman, WA 99164 USA

**Keywords:** Parasitic infection, Proteomic analysis, Confocal microscopy, Antibody generation, ELISA, Immunoblotting, Immunoprecipitation, Cell culture, Flow cytometry, Reverse transcription polymerase chain reaction, Electrophoresis, Mass spectrometry

## Abstract

*Theileria equi* is a widely distributed apicomplexan parasite that causes severe hemolytic anemia in equid species. There is currently no effective vaccine for control of the parasite and understanding the mechanism that *T. equi* utilizes to invade host cells may be crucial for vaccine development. Unlike most apicomplexan species studied to date, the role of micronemes in *T. equi* invasion of host cells is unknown. We therefore assessed the role of the *T. equi* claudin-like apicomplexan microneme protein (CLAMP) in the invasion of equine erythrocytes as a first step towards understanding the role of this organelle in the parasite. Our findings show that CLAMP is expressed in the merozoite and intra-erythrocytic developmental stages of *T. equi* and in vitro neutralization experiments suggest that the protein is involved in erythrocyte invasion. Proteomic analyses indicate that CLAMP interacts with the equine erythrocyte α-and β- spectrin chains in the initial stages of *T. equi* invasion and maintains these interactions while also associating with the anion-exchange protein, tropomyosin 3, band 4.1 and cytoplasmic actin 1 after invasion. Additionally, serological analyses show that *T. equi*-infected horses mount robust antibody responses against CLAMP indicating that the protein is immunogenic and therefore represents a potential vaccine candidate.

## Introduction

*Theileria equi* is an obligate intracellular apicomplexan protist that invades both leukocytes and erythrocytes in mammalian hosts, and causes severe anemia in infected equids, with mortality in some cases^1^. Unfortunately, recovered animals remain persistently infected and are involved in the dynamics of parasite transmission^2^. *T. equi* can be transmitted by multiple ixodidae tick species that thrive in tropical, sub-tropical and temperate climates^1,3^, making control of the global spread of the parasite difficult. Unfortunately, there is of yet no vaccine available for control of *T. equi* and understanding the mechanism that the parasite uses to enter host cells is central to development of an effective vaccine.

*T. equi* is classified within the phylum Apicomplexa due to the presence of secretory organelles on the anterior end of the parasite that are essential for invasion of host cells^4–7^. Key among these organelles are the rod-shaped micronemes, whose contents are first to be discharged following mobilization of intracellular calcium stores^8^, facilitating irreversible attachment of apicomplexan parasites to host cells during invasion^6,9^. Studies have demonstrated a direct link between the number of micronemes and parasite motility and invasion efficacy, since invasive stages of apicomplexan parasites with higher number of micronemes displayed enhanced motility and robust active cell entry^10^ compared to those without, or with fewer, micronemes^9,11^.

Unlike most apicomplexan species, transforming *Theileria* spp*.* developmental stages that invade mammalian cells lack micronemes and are therefore immotile^11,12^. *Theileria parva* (*T. parva*) sporozoites and merozoites for instance interact with host cells by chance and attach irreversibly before utilizing a zippering mechanism to invade the cells passively and in any orientation^12^. By contrast, the non-transforming *T. equi* sporozoites and merozoites contain micronemes^13^. This indicates that the parasite may be motile and utilizes microneme contents to attach to and invade host cells. Unfortunately, this is yet to be ascertained because no studies have been conducted to investigate functional roles of microneme proteins and the mechanism that *T. equi* utilizes to enter host cells.

To clarify this issue, we assessed the role of the claudin-like apicomplexan microneme protein (CLAMP) in invasion of equine erythrocytes, as a first step in dissecting the function of *T. equi* micronemes in host cell invasion. CLAMP was recently identified as an indispensable conserved apicomplexan protein (ICAP) that is conserved in sequence and synteny among apicomplexan species^14^. The study demonstrated that conditional knockdown of the *clamp* gene in *Toxoplasma gondii* and *Plasmodium falciparum* resulted in abrogation of host cell invasion, indicating functional importance of the protein in these species^14^. We hypothesized that like *P. falciparum* and *T. gondii*, *T. equi* expresses CLAMP and uses it during the invasion of equine erythrocytes. The results emerging from this study are consistent with the hypothesis and indicate that CLAMP plays a role in invasion of equine erythrocytes and interacts with key erythrocyte membrane skeleton proteins likely as part of the mechanism of invasion and establishment of the parasite within the cell. The findings also show that CLAMP is considerably immunogenic and is therefore a suitable target for vaccine development.

## Results

### *T. equi* CLAMP is predicted to be an integral membrane protein

**Figure 1:**
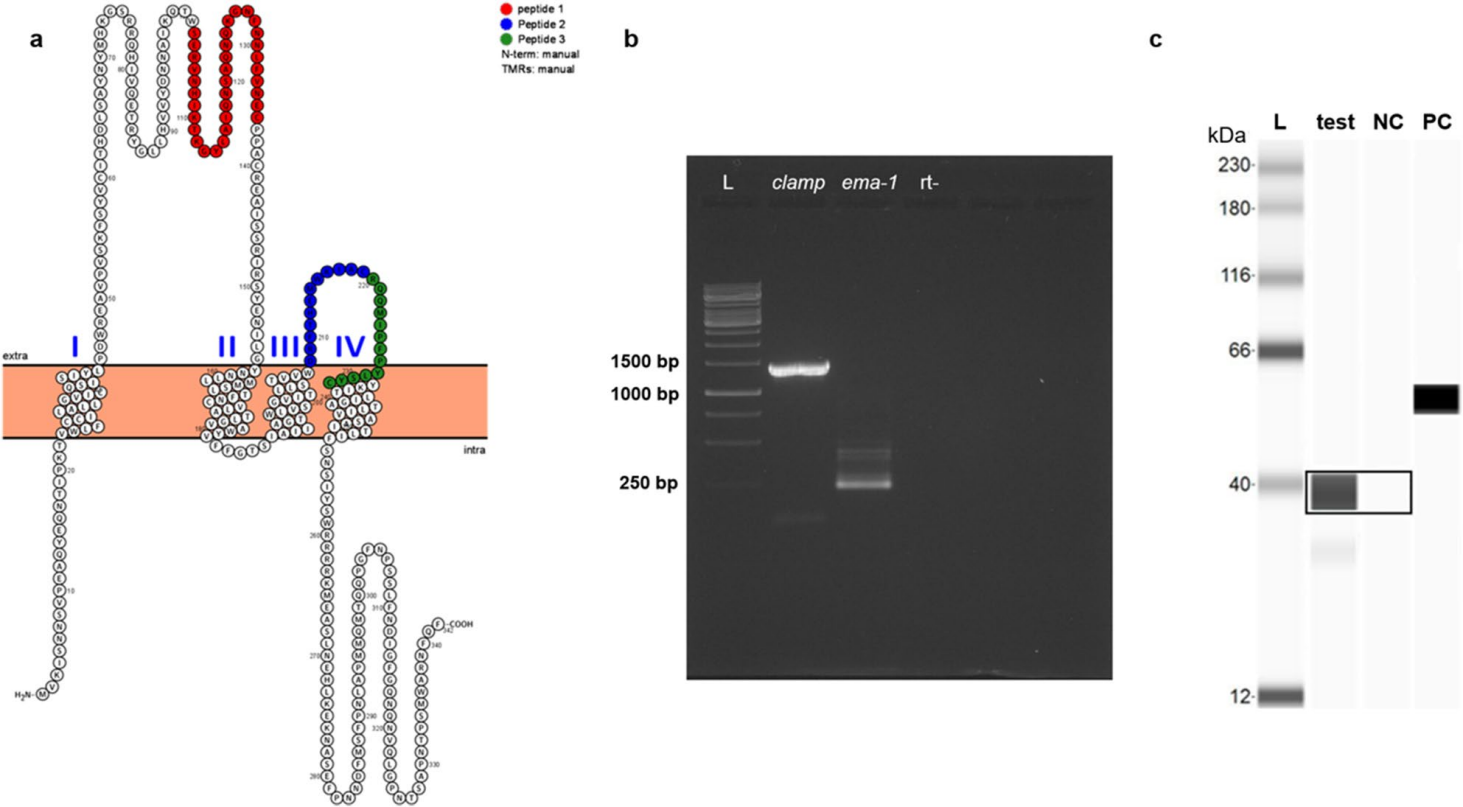
*T. equi* CLAMP contains immunogenic epitopes, and the gene is transcribed and expressed by *T. equi* merozoites. (**a**) CLAMP’s transmembrane topology as predicted and annotated by Phobius and Protter algorithms, respectively. Peptides 1, 2 and 3 indicate the protein’s predicted immunogenic peptides. (**b**) A 1% agarose gel showing amplified *clamp* transcript at ~ 1226 bp. The *T. equi equine merozoite antigen-1* (*ema-1*) transcript was amplified as a positive control, and rt- (negative control) represents amplified merozoites’ RNA without addition of reverse transcriptase. L indicates the Thermo Scientific GeneRuler 1 kb DNA Ladder. (**c**) Immunoblot showing CLAMP expressed by *T. equi* merozoites (test) at ~ 39 kDa. Probing of merozoites lysate with pre-immunization serum and the polyclonal anti-RAP-1a antibody were used as negative control (NC) and positive control (PC) respectively. L represents the ProteinSimple Wes Ladder.

In silico prediction of CLAMP’s transmembrane topology was performed to determine the likelihood that the protein can be recognized by equine immune response components during *T. equi* infection. Phobius (http://phobius.sbc.su.se/) was used to predict CLAMP’s transmembrane profile and Protter^15^ was used to visualize the output. The results showed that *T. equi* CLAMP is likely an integral membrane protein, containing four transmembrane and two extracellular domains (Fig. 1a). In silico B cell epitope mapping was performed for detection of antigenic determinants on CLAMP, and this led to the identification of three potentially immunogenic regions that map onto the two extracellular loops of the protein. Three synthetic peptides representing the regions of the protein that contain predicted B-cell epitopes (Fig. 1a) were synthesized and used to immunize rabbits for development of the polyclonal anti-CLAMP antibody that was used for downstream analyses.

### The *clamp* gene is transcribed, and the protein expressed in merozoites and intra-erythrocytic stages of *T. equi* development

RNA was extracted from *T. equi* merozoites and reverse transcription, cDNA amplification, agarose gel analysis, gel purification and sequencing were performed to assess transcription of the *clamp* gene in the parasite. The results showed that transcription of the gene occurs in merozoites as indicated by the presence of an amplicon of approximately 1226 bp in size (Fig. 1b). Sequencing confirmed that the cDNA amplicon sequence was identical to that available in GenBank (accession number: BEWA_005470).

Immunoblotting was then performed to determine whether merozoites express CLAMP. A *T. equi* merozoite lysate was used as antigen, and the rabbit polyclonal anti-CLAMP antibody developed against the three immunogenic synthetic peptides (Fig. 1a) was used as the primary antibody. The pre-immunization serum and the polyclonal anti-RAP-1a antibody that targets RAP-1a (~ 60 kDa) were used as negative control (NC) and positive control (PC) respectively. The analysis showed that the polyclonal anti-CLAMP antibody recognized a single protein of ~ 39 kDa, which is consistent with the predicted in silico molecular weight of CLAMP (~ 39 kDa), confirming that the protein is expressed by in vitro cultured *T. equi* merozoites (Fig. 1c).

**Figure 2:**
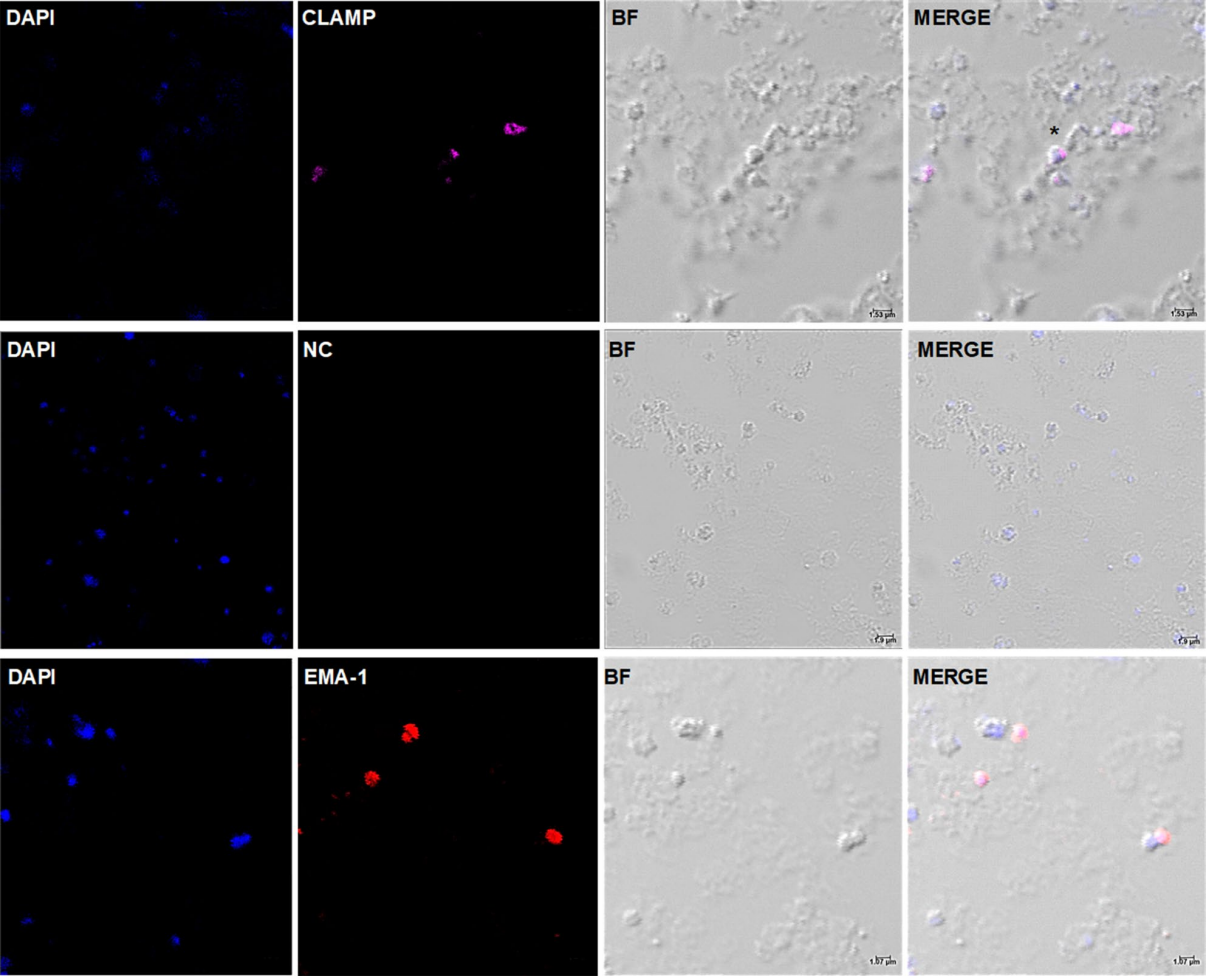
CLAMP is expressed on the surface of *T. equi* merozoites. Confocal microscopy images showing expression of CLAMP by extracellular *T. equi* merozoites. The first row shows expression of CLAMP on the surface of an extracellular merozoite (indicated by an asterisk (*)). The nuclei are stained in blue with DAPI, and CLAMP is stained in magenta. The second row represents merozoites incubated with the pre-immunization serum (negative control (NC)) and shows lack of protein staining indicating that the staining in the first row was CLAMP-specific. The third row shows expression of EMA-1 (positive control (PC)) by merozoites. In all cases, BF refers to brightfield panels.

**Figure 3:**
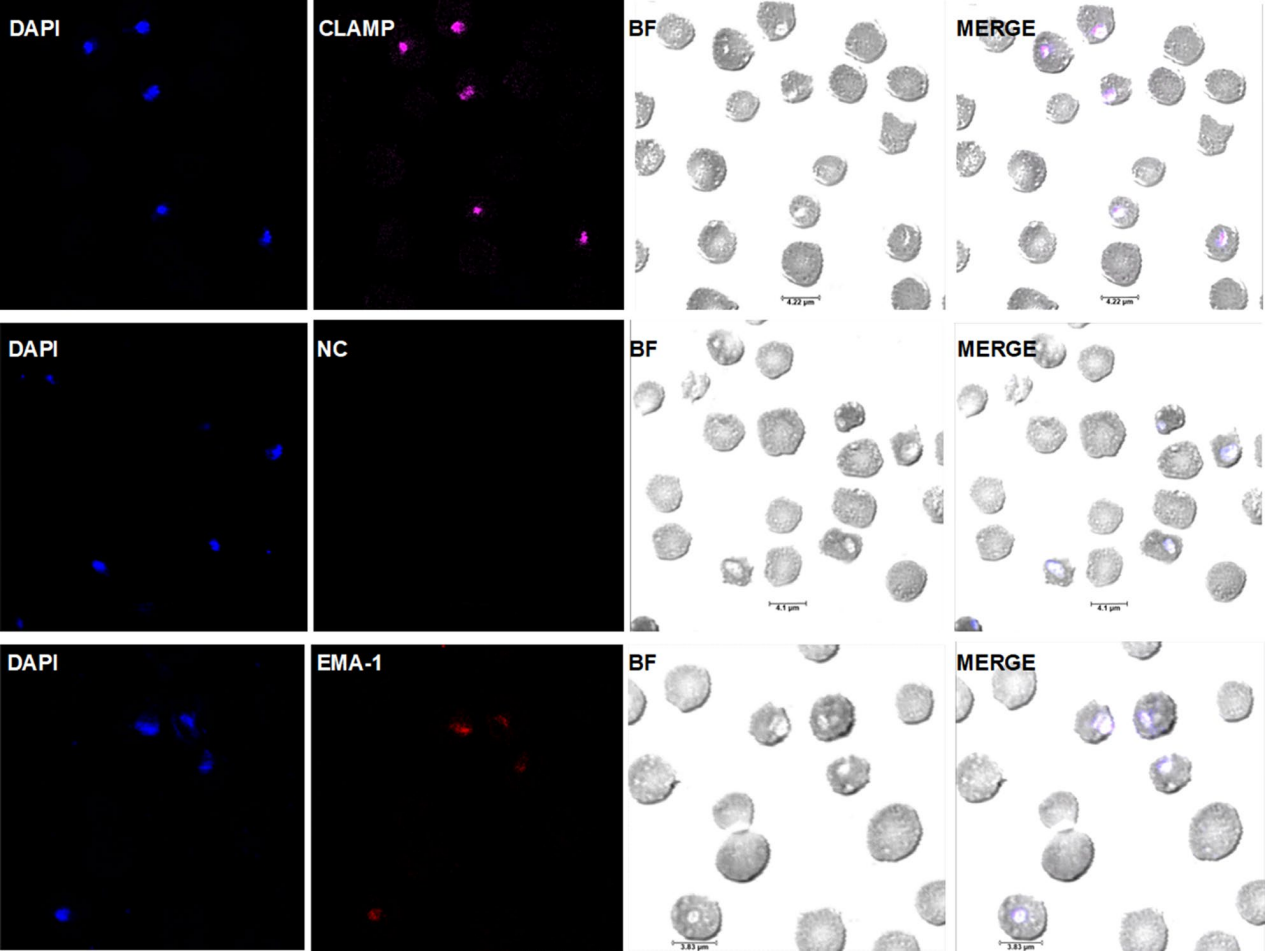
CLAMP is expressed in the intra-erythrocytic stages of *T. equi* development. Confocal microscopy images showing expression of CLAMP in *T. equi* infected erythrocytes. The first row shows expression of CLAMP by *T. equi* gamonts. Parasite nuclei are stained in blue with DAPI, and CLAMP is stained in magenta. The second row shows infected erythrocytes incubated with pre-immunization serum (negative control) and it shows lack of staining indicating test specificity. The third row indicates expression of EMA-1 (positive control (PC)) by intra-erythrocytic parasites. In all cases, BF refers to brightfield panels.

Indirect immunofluorescence antibody tests (IFAT) were conducted to establish whether CLAMP is expressed on the surface of extracellular *T. equi* merozoites and the intra-erythrocytic developmental stages, respectively. In the case of extracellular merozoites, the parasites were incubated with the polyclonal anti-CLAMP antibody prior to incubation with the Invitrogen Goat anti-Rabbit IgG (H + L) Highly Cross-Adsorbed Secondary Antibody, Alexa Fluor Plus 647 and subsequent mounting using the Invitrogen SlowFade Gold Antifade Mountant with DAPI. In the fixed indirect IFAT, fixed slides of *T. equi*-infected erythrocyte smears were incubated with the polyclonal antibody prior to incubation the fluorescent labeled secondary antibody. The resulting confocal microscopy images are consistent with expression of CLAMP on the cell surface of extracellular merozoites (Fig. 2) and in the intra-erythrocytic developmental stages of the parasite (Fig. 3). Incubation of the extracellular parasites and fixed slides with a monoclonal antibody specific for *T. equi* EMA-1 and pre-immunization serum were used as positive and negative controls, respectively.

### CLAMP elicits antibody responses in *T. equi* infected horses

**Figure 4:**
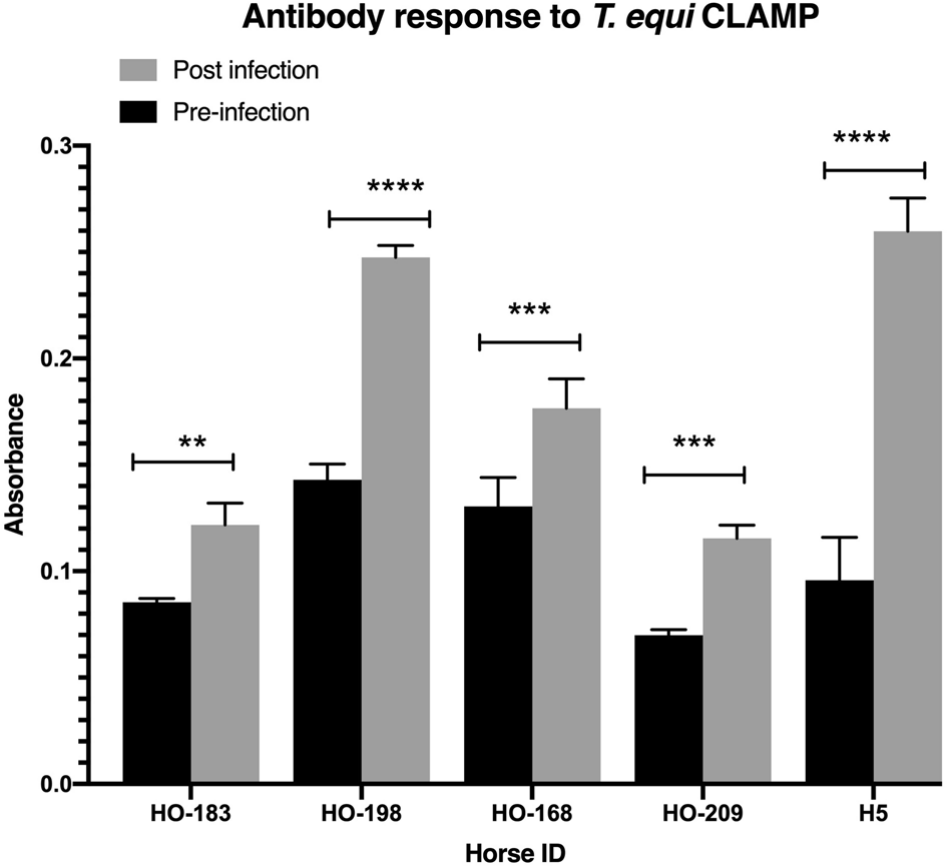
CLAMP elicits antibody responses in horses during infection with *T. equi*. CLAMP-specific antibodies are significantly present in horses after infection with *T. equi* (post- infection). **p = 0.0014, ***p = 0.0002, ****p < 0.0001.

Serological analysis using an indirect ELISA was performed to determine whether CLAMP elicits antibody responses in *T. equi*-infected horses. A cocktail of the three synthetic CLAMP peptides containing predicted B-cell epitopes (Fig. 1a) was used as the antigen and sera obtained from five horses i.e., H5, HO-209, HO-168, HO-198 and HO-183 prior to *T. equi* infection (pre-infection) were used as negative controls. Sera obtained from the horses at 1.5 to 34 months post-infection were used to assess development of antibody responses to CLAMP during *T. equi* infection. Two-way ANOVA was used for statistical analysis at a significance level (α) of 0.05, and the results showed that the *T. equi* infected horses developed significant antibody responses against the synthetic peptides that represent predicted B-cell epitopes of CLAMP (Fig. 4).

### CLAMP-specific antibodies inhibit invasion of equine erythrocytes by *T. equi*

**Figure 5:**
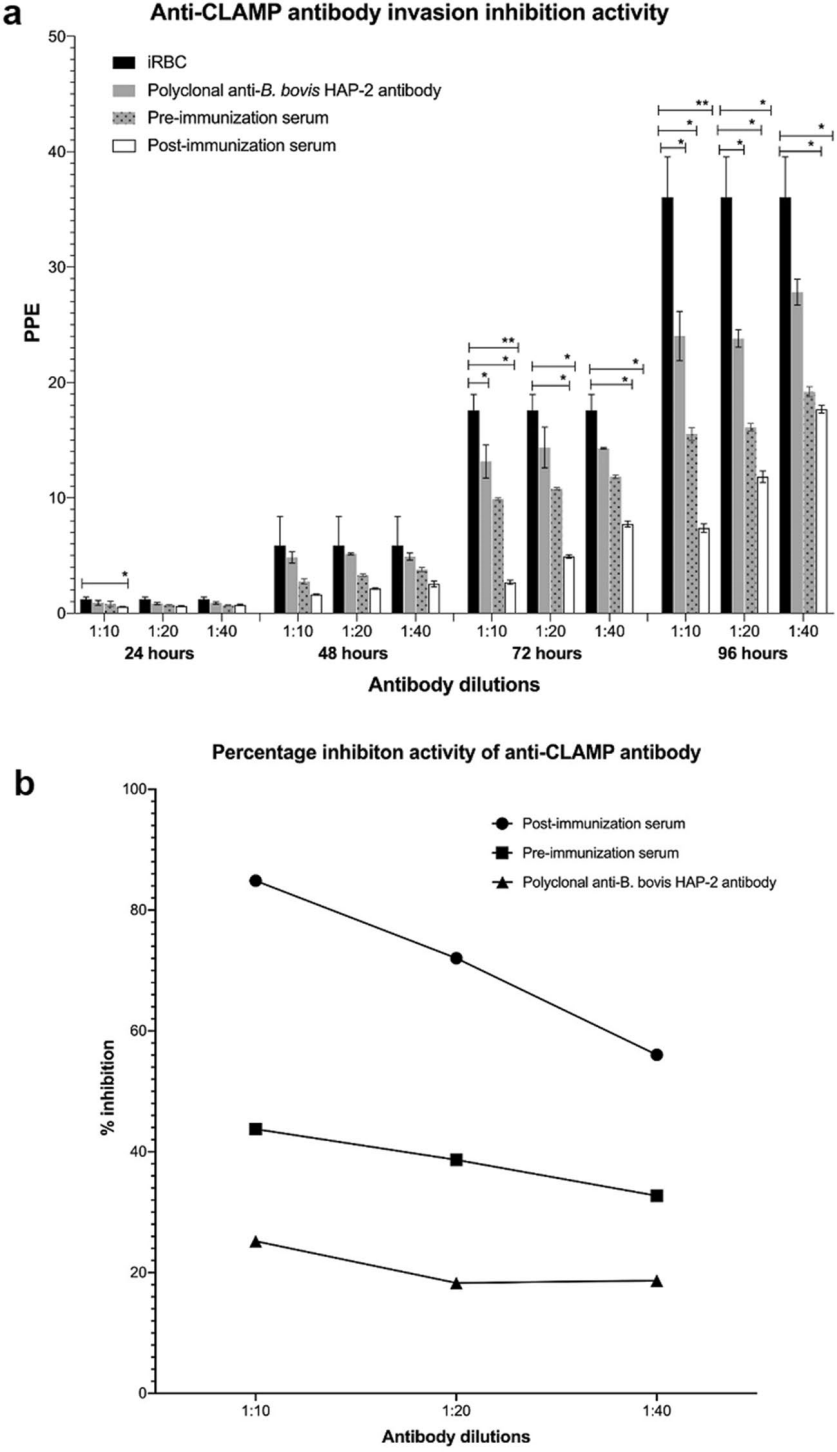
Polyclonal anti-CLAMP antibody significantly inhibits invasion of equine erythrocytes by *T. equi*. (**a**) In vitro neutralization assay data showing invasion inhibition activity of the CLAMP-specific antibody (post-immunization serum), pre-immunization serum and polyclonal anti-*B. bovis* HAP-2 antibody at different dilutions over time. *T. equi* infected erythrocytes cultured in the presence of CLAMP-specific antibodies were significantly less parasitized (PPE) compared to parasites cultured in the presence of pre-immunization serum and the polyclonal anti-*B. bovis* HAP-2 antibody. Control represents *T. equi* infected red blood cells (iRBC) cultured in the absence of both pre- and post-immunization sera. *p < 0.05, **p < 0.0090. (**b**) Anti-CLAMP antibodies inhibit a significant percentage of parasites from invading erythrocytes at 72 h post-infection. Percentage inhibition was calculated as the difference between percentage inhibition in the presence of polyclonal anti-CLAMP antibodies (post-immunization serum) and percentage inhibition in the presence of pre-immunization serum. In this regard, anti-CLAMP antibodies in the post-immunization serum inhibited parasite invasion by 41%, 33.4% and 23.3% at 1:10, 1:20 and 1: 40 dilutions, respectively. Factors present in the *B. bovis* HAP-2 post immunization serum (polyclonal anti-*B. bovis* HAP-2 antibody) also minimally inhibited invasion.

An in vitro neutralization assay was performed to determine whether neutralization of CLAMP by specific antibodies prevents *T. equi* from invading equine erythrocytes. *T. equi*-infected cells were separately incubated with heat-inactivated pre-immunization serum, post-immunization serum (polyclonal anti-CLAMP antibody) and polyclonal anti-*B. bovis* HAP-2 antibody. The sera were diluted in *T. equi* growth medium at 1:10, 1: 20 and 1:40 dilutions. Each dilution was assessed in triplicate, and the cultures were harvested at 24, 48, 72- and 96-h post-infection. Flow cytometry was performed to evaluate the degree of invasion inhibition in the presence of post-immunization and pre-immunization sera relative to infected erythrocytes cultured in the absence of both sera (control). Statistical analysis was performed using two-way ANOVA at a significance level of 0.05, and it was evident that addition of polyclonal anti-CLAMP antibody to the cultures results in significant inhibitory effect on erythrocyte invasion by *T. equi* over time. Conversely, the pre-immunization serum and polyclonal anti-*B. bovis* HAP-2 antibody did not significantly inhibit parasite invasion. This indicated that the inhibitory effect was likely specific to the anti-CLAMP antibody (Fig. 5a).

Percentage inhibition capacity for each dilution of the polyclonal anti-CLAMP antibody was determined at 72 h post *T. equi* infection because optimum neutralization activity of the antibody was observed at this time point. The results were compared to percentage inhibition in the presence of pre-immunization serum, and the output showed that incubation of *T. equi* merozoites using the optimal antibody dilution (1:10) inhibited parasite invasion by 85%, while incubation with the pre-immunization serum at the same concentration inhibited invasion by 44% (Fig. 5b). This strongly suggested that serum factors other than CLAMP-specific antibodies may also contribute to invasion inhibition. We therefore concluded that the difference between the two percentages (i.e., 41%) represented the actual percentage inhibition value of CLAMP-specific antibodies at 1:10 dilution. Percentage inhibition analysis showed that the polyclonal anti-*B. bovis* HAP-2 antibody also minimally inhibited invasion confirming that other factors present in the immune serum may contribute to neutralization of parasite invasion.

Collectively, these findings indicate that CLAMP is involved in invasion of equine erythrocytes.

### *T. equi* CLAMP interacts with components of the equine erythrocyte membrane skeleton

Cross-linking of equine erythrocyte extracellular, intramembrane, and intracellular proteins to *T. equi* CLAMP was performed prior to co-immunoprecipitation to isolate proteins that interact with CLAMP. SDS-PAGE analysis was performed for detection of CLAMP’s interacting partners, and western blot analysis was conducted to confirm that CLAMP was isolated alongside its interacting partners.

**Figure 6:**
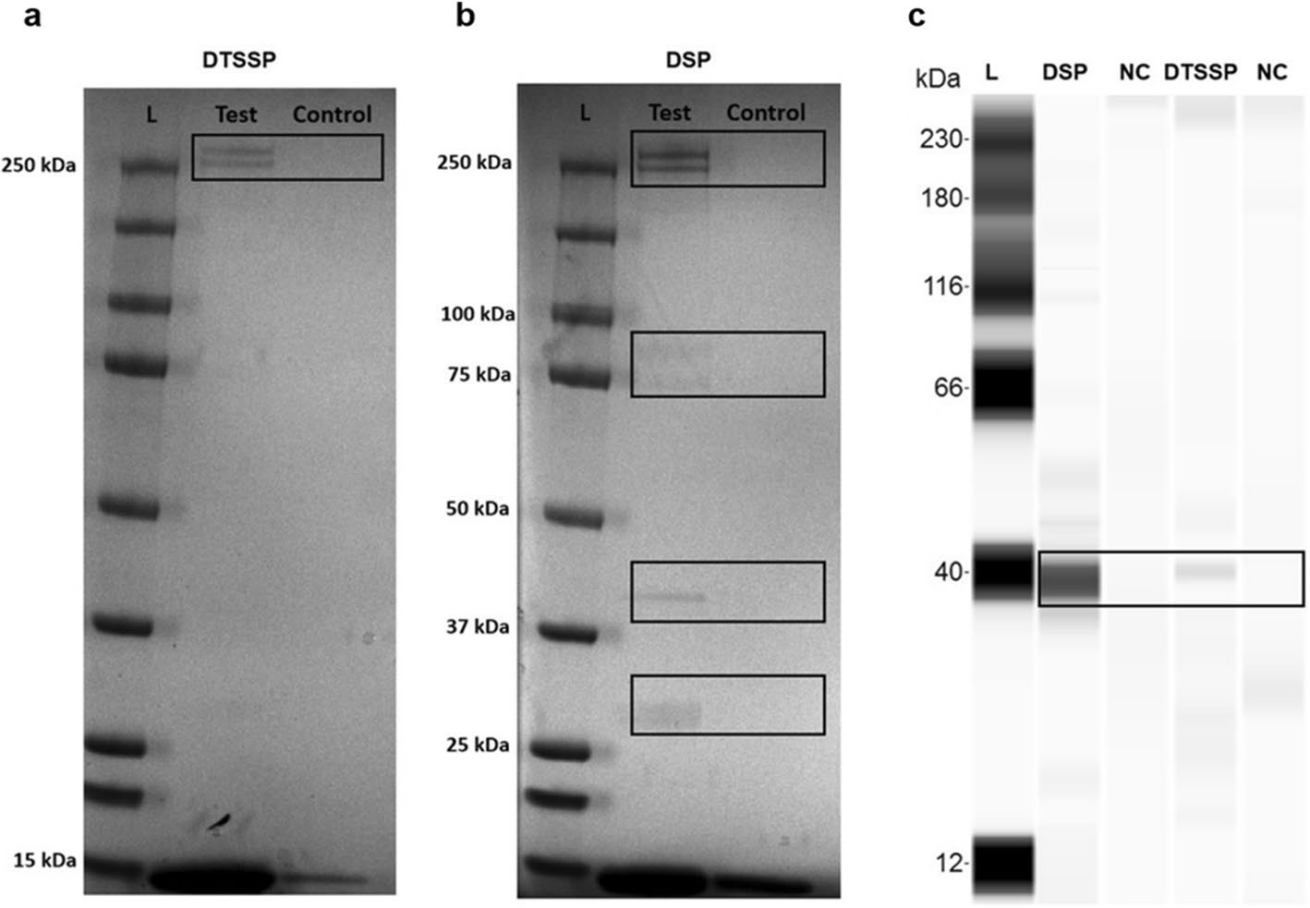
*T. equi* CLAMP interacts with equine erythrocyte proteins. (**a**,**b**) show SDS-PAGE gels of proteins (within black boundaries) isolated from the erythrocyte surface (test) and within the cell membrane and cell (test) after crosslinking to CLAMP with DTSSP and DSP, respectively. (**c**) Represents an immunoblot showing the presence of CLAMP at ~ 39 kDa. In all cases agarose resin eluates were used as negative controls (control/NC). The ~ 15 kDa band on the SDS-PAGE gels indicate non-specific binding of the equine α- and β-hemoglobin subunits to the Thermo Scientific AminoLink (test) and agarose (control) coupling resins.

SDS-PAGE analysis showed the presence of two high molecular weight bands (> 250 kDa) after crosslinking with the 3,3´-dithiobis (sulfosuccinimidylpropionate) (DTSSP) crosslinker that targets extracellular proteins (Fig. 6a). Several bands were observed after crosslinking with the dithiobis (succinimidylpropionate) (DSP) crosslinker that targets intramembrane and intracellular proteins (Fig. 6b). Western blot analysis showed the presence of an approximately 39 kDa band confirming that CLAMP was eluted alongside its interacting partners both on the surface and within equine erythrocytes (Fig. 6c).

Nano LC–MS/MS was performed to identify the equine erythrocyte proteins that interact with CLAMP. The resultant data revealed that the protein interacts with key components of the equine erythrocyte membrane skeleton^16^ including the α- and β- spectrin chains, anion-exchange protein, band 4.1, cytoplasmic actin 1 and tropomyosin 3 (Table 1 and Supplementary Table 1).

**Table 1:** CLAMP interacts with components of the erythrocyte membrane skeleton in the inner cell membrane of equine erythrocytes.

Crosslinker	Protein ID	Uniprot accession number	Molecular weight (KDa)	Erythrocyte compartment
DTSSP	Spectrin alpha, erythrocytic 1	F6ZK25	280.9	Inner cell membrane
	Spectrin beta chain	F6SIV4	268.2	
DSP	Spectrin alpha, erythrocytic 1	F6ZK25	280.9	
	Spectrin beta chain	F6SIV4	268.2	
	Anion—exchange protein	Q2Z1P9	104.2	
	Band 4.1	A0A3Q2H952﻿	110.4	
	Actin, cytoplasmic 1	F6T3Y8	41.9	
	Tropomyosin 3	A0A5F5PGV6	28.9	

## Discussion

The prediction of B-cell epitopes on the extracellular loops of *T. equi* CLAMP suggested that the protein is expressed on the cell surface. This in silico prediction suggests that CLAMP could be a microneme-specific protein that is translocated to the cell surface rendering it possible for it to be recognized by protective equine immune responses during *T. equi* infection. This was consistent with the production of robust CLAMP-specific antibodies in horses during infection with the parasite, indicating that the protein is immunogenic, and is therefore a suitable candidate for evaluation as a vaccine component.

The indirect immunofluorescence antibody tests further suggests that translocation of CLAMP occurs from the micronemes to the cell surface in extracellular merozoites, and its expression is maintained in the intra-erythrocytic developmental stages. This indicates functional significance of the protein in the parasite. A significant role of *T. equi* CLAMP in invasion of equine erythrocytes was demonstrated by the in vitro neutralization assay, which showed that CLAMP-specific antibodies significantly inhibit cellular invasion by merozoites. This finding also suggests that the antibodies developed against CLAMP in vivo could also have a neutralizing capacity against *T. equi*, thus reinforcing the fact that the protein is a viable vaccine candidate and future studies should be focused on testing its potential in vivo.

Investigation of erythrocyte proteins that interact with CLAMP presumably during invasion and establishment of *T. equi* within the cell led to the isolation and identification of key components of the erythrocyte membrane skeleton that form a complex network in the inner membrane of the cell^16^. These proteins maintain the structure, shape, durability, stability, and plasticity of the cell membrane^16,17^. Crosslinking of CLAMP to extracellular erythrocytic proteins led to isolation of the α- and β-spectrin proteins and crosslinking of the protein to intramembrane and intracellular erythrocytic proteins led to the isolation of the spectrin proteins alongside the anion-exchange protein, band 4.1, cytoplasmic actin 1 and tropomyosin 3.

**Figure 7:**
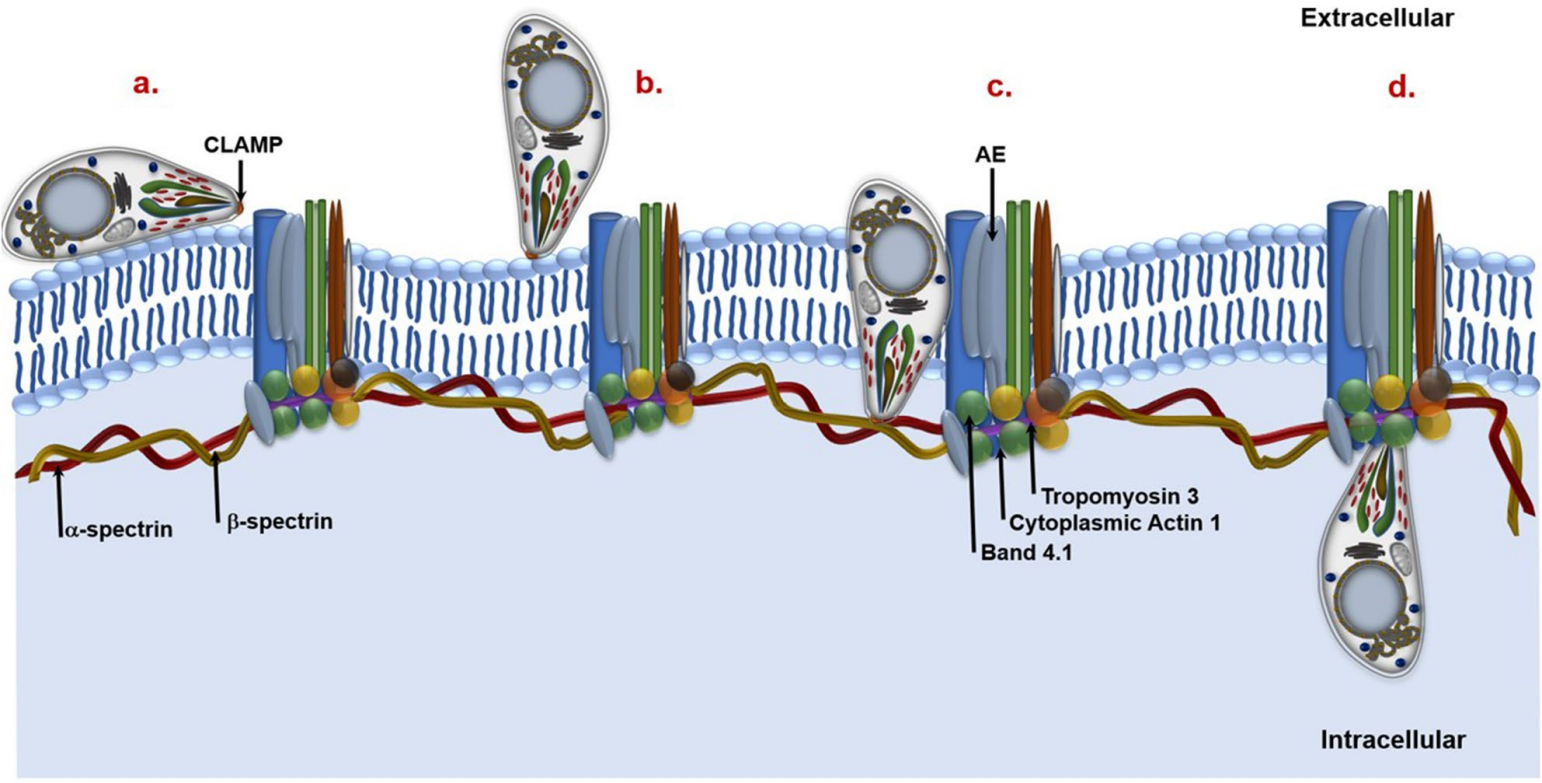
Hypothetical model showing the role of *T. equi* CLAMP in invasion of equine erythrocytes and its interactions with membrane skeleton proteins. (**a**) *T. equi* attaches to the equine erythrocyte surface, and (**b**) reorientates itself such that the apical end is in direct association with the cell membrane. (**c**) The parasite uses CLAMP (that is possibly translocated from the micronemes to the cell surface) to attach to the α- and β-spectrin proteins during the initial stages of invasion. (**d**) Once inside the cell, *T. equi* CLAMP maintains its interactions with the spectrin proteins while also interacting with band 4.1, tropomyosin 3, cytoplasmic actin 1 and anion-exchange protein (AE).

A model emerging from the interpretation of these findings suggest that *T. equi* merozoites use CLAMP to attach to the α- and β-spectrin proteins in the initial stages of invasion, possibly during the formation of a tight junction between the parasite and the host cell. Once inside the cell, the parasites maintain their association with the spectrin proteins while also interacting with the anion-exchange protein, band 4.1, cytoplasmic actin 1 and tropomyosin 3 (Fig. 7). This observation indicates that like most apicomplexan species, the early stages of *T. equi* invasion is possibly characterized by attachment and re-orientation of the parasite such that the anterior end is in direct apposition with the host cell^6,7^. This enables CLAMP to interact with the spectrin proteins in the inner cell membrane and these interactions are maintained post-invasion (Fig. 7). This suggests that *T. equi* micronemes are involved in the invasion of equine erythrocytes thus implying that the parasites utilize a mechanism that is distinct from that of transforming *Theileria* spp*.*^12^ to enter erythrocytes. Notably, a study conducted by Fawcett et al. showed that *T. parva* develops micronemes in the tick salivary gland during sporogony^18^. This indicates that the parasite possibly undergoes morphological changes through the life cycle and microneme-specific proteins could be important in the salivary gland developmental stage. Whether CLAMP is also involved in the invasion of leukocytes by *T. equi* sporozoites remains unknown.

Previous studies have shown that *P. falciparum* exploits components of the erythrocyte membrane skeleton to maintain itself in infected cells. For instance, the parasite utilizes the ring-infected erythrocyte surface antigen (RESA) that is expressed in the early intra-erythrocytic stages to bind to spectrin^19^, rendering the cell resistant to thermal stress during febrile conditions^20,21^ and resilient to mechanical pressure^22^. The interaction also prevents other parasites from invading parasitized cells thus minimizing the number of *P. falciparum* in a single cell^22^. Conversely, the interaction between α-spectrin and the *P. falciparum* erythrocyte membrane protein 3 (PfEMP3) that is expressed in the late stages of intra-erythrocytic development, leads to disruption of the spectrin-actin-4.1R network rendering the cell susceptible to mechanical pressure^23^. It is suspected that this interaction contributes to egress of mature merozoites from infected erythrocytes^23^. Studies have also shown that the cytoplasmic domain of the *P. falciparum* erythrocyte membrane protein 1 (PfEMP1) interacts with spectrin, actin and the *P. falciparum* knob-associated histidine-rich protein (KAHRP)^24^. These interactions anchor PfEMP1 to the erythrocyte membrane allowing it to mediate adhesion of infected erythrocytes to receptors on endothelial cells^25^. Additionally, Magowan et al. showed that binding of the mature-parasite-infected erythrocyte surface antigen (MESA) to protein 4.1 is crucial for the intra-erythrocytic survival of *P. falciparum*^26^.

Collectively, these studies show that the interaction between parasite and host erythrocyte membrane skeleton proteins is important for the survival of *P. falciparum* in infected erythrocytes, suggesting that this may also be the case for *T. equi*. Further studies are therefore required to determine whether the role of CLAMP extends beyond the erythrocyte entry process and to ascertain whether it interacts with the entire erythrocyte membrane protein complex or primarily with one of the network of co-immunoprecipitated host proteins.

In conclusion, the immunogenicity of CLAMP and neutralization capacity of CLAMP-specific antibodies suggest that the protein can be explored for development of an effective vaccine against *T. equi* and other apicomplexan parasites because it is conserved among these species^14^. Our discovery of the importance of CLAMP, and by implication also micronemes, in the invasion of equine erythrocytes by *T. equi* provides further evidence that in addition to their intermediate position between *Babesia* and *Theileria,* based on comparative genomics^27,28^, *Theileria* spp. that only transiently infect host leukocytes without inducing immortalization are also distinct in their cell biology relative to transforming *Theileria*. The identification of equine erythrocyte proteins that interact with CLAMP highlight dynamics of host-parasite interactions between *T. equi* and the host cells. Further studies are required to not only establish the significance of these interactions in invasion of erythrocytes and parasite survival, but to also determine how the interactions can be applied in drug discovery studies for the control of *T. equi*.

## Methods

### Ethics statement

All animal experiments were approved by the Institutional Animal Care and Use Protocol Committees of the Pacific Immunology Corporation, CA, USA (protocol #11/11/19. Ref. SOP-1) and the University of Idaho, ID, USA (protocol #2010-54). The procedures were performed according to the U.S. National Institutes of Health (NIH) Guide for the Care and Use of Laboratory Animals and the ARRIVE guidelines and regulations.

### Experimental animals

Four New Zealand white rabbits (14481, 14482, 14483 and 14484) and five Welsh cross horses (H5, Te0018 (HO-168), HO-198, HO-209 and HO-183).

### Parasite

The *T. equi* Florida strain was used in the experiments described herein.

### In silico analysis

The *T. equi* CLAMP amino acid sequence (accession number: BEWA_005470) was retrieved from GenBank. Phobius (http://phobius.sbc.su.se/) was used to predict the protein’s transmembrane topology and Protter^15^ was used for visualization and annotation of the transmembrane profile. B cell epitope mapping was performed using a proprietary suite of 20 separate predictive algorithms available at Pacific Immunology Corporation. This led to the identification of three immunogenic peptides i.e. peptide 1 (SERVNHIKTKGYLAIQNSAQNQKGNFNNLFVNEC), peptide 2 (DRFTHEMWKIAC) and peptide 3 (RQQMIPFPYLSYC) that map onto positions 103–136, 208–219 and 220–232 of the CLAMP amino acid sequence, respectively. The peptides were synthesized by Pacific Immunology Corporation.

### Generation of *T. equi*-infected erythrocyte cultures and isolation of extracellular merozoites

*T. equi*-infected erythrocyte cultures were set up in five 25 cm^2^ Corning cell culture flasks and maintained as previously described^29^ until a parasitemia of ~ 10% was attained. To isolate extracellular merozoites, the infected cell cultures were centrifuged at 650*xg* for 10 min to pellet erythrocytes. The supernatant was obtained and spun at 2800*xg* for 30 min at 10 °C to pellet merozoites. The parasites were washed twice with 40 ml of 1X phosphate buffered saline (PBS) (pH7.2) and suspended in 10 ml of the same buffer.

### Transcriptional analysis of the *clamp* gene in *T. equi* merozoites

RNA was extracted from *T. equi* merozoites using the Qiagen RNeasy Mini Kit (Qiagen, Hilden, Germany) in compliance with the manufacturer’s instructions. Residual DNA was removed using the Invitrogen DNase I, Amplification Grade Kit (Thermo Fisher Scientific, Waltham, MA) and cDNA synthesis was performed using the Invitrogen SuperScript III First-Strand Synthesis System in accordance with the manufacturer’s instructions. Amplification of the transcript was performed using the Invitrogen Platinum SuperFi DNA Polymerase with specific primers (*clamp* fwd; 5′-GTA TAC ACA GAT AAG CCA TAA ATA ATC GTG-3′ and *clamp* rev; 5′-TCA AAA CTG GAA GTT ACG TGC C-3spp′). The PCR conditions used were as follows; initial denaturation at 98 °C for 30 s, 40 cycles of denaturation at 98 °C for 10 s, annealing at 57.6 °C for 30 s and extension at 72 °C for 90 s. Final extension was conducted at 72 °C for 10 min and transcription of the *clamp* gene was visualized using a 1% agarose gel. The Qiagen QIAquick Gel Extraction Kit was used to clean-up the amplified transcript in accordance with the manufacturer’s instructions and Sanger sequencing was performed to confirm the integrity of the amplicon. Amplification of the *equine merozoite antigen-1* (*ema-1*) transcript as previously described^30^ was used as a positive control, and amplification of RNA without addition of reverse transcriptase was used as a negative control.

### Evaluation of expression of CLAMP in merozoites and intra-erythrocytic stages of *T. equi* development

#### Development of polyclonal antibody against synthetic CLAMP peptides

The polyclonal anti-CLAMP antibody was produced by Pacific Immunology Corporation (Pacific Immunology, Ramona, CA). Briefly, 5 ml of serum (pre-immunization serum) was obtained from each of the New Zealand white rabbits prior to immunization with 1 mg/ml of synthetic CLAMP peptides conjugated to the keyhole limpet hemocyanin (KLH) carrier protein. The peptide conjugates were mixed with the Pacific Immunology AdjuLite Freund’s complete adjuvant at a 1:1 ratio. Rabbits 14481 and 14482 were immunized with peptide 1 and rabbits 14483 and 14484 were co-immunized with peptides 2 and 3. Three subsequent immunizations were performed at a 21-day interval, with the synthetic peptide conjugates mixed with the Pacific Immunology AdjuLite Freund’s incomplete adjuvant at a 1:1 ratio. 25 ml of serum was collected from the rabbits at 49- and 63-days post-initial immunization and at 7- and 21-days post-final boost to assess antibody responses. The rabbits were euthanized at 31 days post-final boost, and serum containing the polyclonal antibody was obtained from the individual rabbits and pooled for downstream analysis.

#### Assessment of the expression of CLAMP in extracellular T. equi merozoites

Immunoblotting was performed to evaluate expression of CLAMP in extracellular *T. equi* merozoites. The ProteinSimple Wes Kit (ProteinSimple, San Jose, CA) was used to conduct the analysis in accordance with the manufacturer’s instructions. Merozoite lysate was used as the antigen and the rabbit polyclonal anti-CLAMP antibody was used as a primary antibody at 1:100 dilution. The KPL affinity purified peroxidase labeled Goat anti-Rabbit IgG (H + L) antibody (Seracare Life Sciences, Milford, MA) was used as a secondary antibody at 1: 500 dilution. In the case of negative and positive controls, merozoite lysates were used as the antigen and pre-immunization serum and the polyclonal anti-RAP-1a antibody were used as primary antibodies at 1:100 and 1:250 dilutions, respectively. A chemiluminescent detection method using luminol and peroxide was used for band detection, and the Simple Western Wes capillary-based immunoassay platform (ProteinSimple, San Jose, CA) was used to perform the protein separation. The Compass software was then used for detection and output analysis.

An indirect immunofluorescence antibody test with subsequent fixation and permeabilization was performed to confirm that CLAMP is expressed on the surface of *T. equi* as predicted by in silico analysis. Briefly, 6-CFDA staining was performed as previously described^31^ for viability assessment and quantification of the extracellular merozoites. 1 ml of 1X PBS containing viable 2.5 × 10^8^ merozoites was then pipetted into three 1.5 ml Eppendorf tubes. The tubes were spun at 3000*xg* for 5 min to pellet the parasites. The merozoites were obtained and incubated with 10% bovine serum albumin (BSA) in 1X PBS (blocking buffer) at room temperature for 15 min. The parasites were pelleted and washed twice using 500 µl of 1XPBS prior to being incubated with the polyclonal anti-CLAMP antibody diluted in the blocking buffer at 1:100 (test sample). Monoclonal anti-EMA-1 antibody and the pre-immunization serum were used as positive and negative controls at 1:200 and 1:100 dilutions, respectively. Incubation was performed at 37 °C for 30 min and the parasites were spun and washed thrice prior to incubation with fluorescent-labeled secondary antibodies. The test sample and negative control were incubated with the Invitrogen Goat anti-Rabbit IgG (H + L) Highly Cross-Adsorbed Secondary Antibody, Alexa Fluor Plus 647 and the positive control was incubated with Invitrogen Goat anti-Mouse IgG (H + L) Highly Cross-Adsorbed Secondary Antibody, Alexa Fluor 594. Both antibodies were diluted in the blocking buffer at 1:200 and incubation was performed at 37 °C for 30 min prior to being pelleted once more and washed thrice. The merozoites were suspended in 50 µl of 1X PBS and 10 µl of the suspensions from each of the Eppendorf tubes were transferred onto respective wells of a 12-well teflon printed diagnostic microscopic slide. The slide was air-dried and fixed using ice cold acetone and methanol as previously described^32^. It was then air-dried, and mounting was performed using the Invitrogen SlowFade Gold Antifade Mountant with DAPI. A coverslip was used to cover the slide and confocal microscopy was performed using the Leica microsystems SP8-X white light pulsed laser point scanner with lightning confocal microscope (Leica Microsystems Inc., Buffalo Grove, IL) and the data was collected using the Leica Application Suite X (LAS X) software.

#### Evaluation of the localization and expression of CLAMP in T. equi’s intra-erythrocytic developmental stages

The indirect fixed immunofluorescence antibody test was performed to assess expression of CLAMP in the intra-erythrocytic stages of *T. equi* development. Briefly, infected erythrocyte smears were permeabilized and fixed using ice cold acetone and methanol at equal ratios as previously described^32^. The slides were air dried and blocked with 200 µl of the blocking buffer for 15 min at 37 °C in a wet chamber prior to being washed twice with 1X PBS. 200 µl of the polyclonal anti-CLAMP antibody diluted in the blocking buffer at 1:10 dilution was pipetted onto one of the slides (test slide). 1:10 and 1:50 dilutions of the pre-immunization serum and monoclonal anti-EMA-1 antibody were used as negative and positive controls, respectively. The slides were incubated at 37° C for 30 min prior to being washed twice with 1X PBS. The test and negative control slides were then incubated with 200 µl of the Invitrogen Goat anti-Rabbit IgG (H + L) Highly Cross-Adsorbed Secondary Antibody, Alexa Fluor Plus 647 diluted in the blocking buffer at 1:200. The positive control was incubated with 200 µl of the Invitrogen Goat anti-Mouse IgG (H + L) Highly Cross-Adsorbed Secondary Antibody, Alexa Fluor 594 diluted at 1:200. The three slides were incubated at 37 °C for 30 min prior to being washed twice using 1X PBS. They were then air-dried, and the Invitrogen SlowFade Gold Antifade Mountant with DAPI was used for mounting. Evaluation of the localization and expression of CLAMP was performed using the Leica microsystems SP8-X white light pulsed laser point scanner with lightning confocal microscope and LAS X software was used for data acquisition.

### Assessment of antibody responses to CLAMP in *T. equi* infected horses

Indirect ELISA was conducted to determine whether CLAMP elicits antibody responses in *T. equi* infected horses. Horse sera obtained from previous studies were used in this analysis. This included sera from horses H5^33^, Te0018 (HO-168)^34^ and HO-198, HO-209 & HO-183^35^ that were collected prior to *T. equi* infection and at 34, 14, 11, 1.5 and 18 months post-infection respectively. Briefly, two 96-well Thermo Scientific Nunc, Immulon plates (Thermo Fisher Scientific, Waltham, MA) were labeled as pre- and post-infection plates. The wells were then coated with 100 µl/ well of the combined CLAMP synthetic peptides diluted in 0.05 M carbonate-bicarbonate (Na_2_CO_3_-NaHCO_3_) buffer (pH 9.6) to final concentrations of 0.05 mg/ml each. Coating was performed in triplicates and the plates were incubated overnight at 4 °C. They were then washed once using 0.05% tween 20 diluted in 1X PBS (wash buffer) and blocked with 200 µl of 20% skimmed milk diluted in the wash buffer at room temperature for 1 h. The blocking buffer was discarded and 75 µl of the pre- and post-infection sera diluted in 0.3% BSA (diluted in the wash buffer) were pipetted into wells of the respective plates at 1:100 dilution. The plates were incubated at room temperature for one hour prior to being washed four times. 100 µl of the KPL affinity purified peroxidase labeled goat anti-Rabbit IgG (H + L) antibody diluted in the dilution buffer at 1:10,000 was pipetted into the wells, and the plates were incubated at room temperature for one hour. The wells were then washed four times and 100 µl of the Thermo Scientific 1-Step Ultra TMB-ELISA substrate solution was added to each well. Incubation was performed at room temperature for 15 min prior to addition of 100 µl of 2 M sulfuric acid to stop the reaction. The SpectraMax 190 microplate reader (Molecular Devices, San Jose, CA) was used to measure the absorbance of each well at 450 nm. Cut-off was calculated using the formula: mean (of the pre-infection serum) + 3*SD (of the pre-infection serum)^36^. Any value above the cut-off was considered to be a true positive. Two-way ANOVA available in GraphPad Prism 8.4.3 (GraphPad Software, San Diego, CA) was used to perform the statistical analysis at a significance level (α) of 0.05.

### Evaluation of the role of *T. equi* CLAMP in the invasion of equine erythrocytes

The in vitro neutralization assay was performed to determine whether *T. equi* utilizes CLAMP to invade host cells. Briefly, heat inactivated post-immunization serum (polyclonal anti-CLAMP antibody) was diluted in 162 µl of the *T. equi* growth medium^29^ at 1:10, 1:20 and 1:40 dilutions prior to being added to the wells of a Corning Costar 96-well flat-bottom tissue culture-treated plate (Millipore Sigma, Burlington, MA) in triplicate. Heat inactivated pre-immunization serum and polyclonal anti-*B. bovis* HAP-2 antibody diluted in *T. equi* growth medium at dilutions identical to the polyclonal anti-CLAMP antibody were used as controls to evaluate test specificity. Like *T. equi* CLAMP, the polyclonal anti-*B. bovis* HAP-2 antibody was produced by immunizing New Zealand white rabbits with synthetic HAP-2 peptides^37^ conjugated to KLH and in the presence of the AdjuLite Freund’s adjuvants. 18 µl of *T. equi* infected erythrocytes at 0.2% parasitemia were added to the wells and the plate was incubated at 37 °C and 5% CO_2_ for 96 h. The antibody containing medium was replaced after every 24 h.

5 µl of the infected cells were harvested from the bottom of the wells after every 24 h and centrifuged at 500*xg* for 5 min at 4 °C. The pelleted cells were obtained and washed twice with 200 µl of 1X PBS (pH 7.2) prior to being suspended in 100 µl of the same buffer containing 25 µg/µl of the Invitrogen Dihydroethidium to stain the parasites’ nuclei. The cell suspension was incubated at 5% CO_2_ and 37 °C for 30 min in the dark prior to being washed twice with 200 µl of 1X PBS. They erythrocytes were then suspended in 200 µl of the same buffer.

Flow cytometry was performed to assess the neutralization capacity of the polyclonal anti-CLAMP antibody as previously described^38^. Briefly, the suspended cells were analyzed by the Guava easyCyte flow cytometer (Luminex Corporation, Austin, Tx) at a proportion of 800–1,000 cells/µl with 20,000 events acquired. Normal, uninfected equine erythrocytes (nRBC) and infected erythrocytes cultured in the absence of antibodies (iRBC) were used as negative and positive controls respectively in the flow cytometric analysis. The results were analyzed using the De Novo FCS Express v6 software (De Novo Software, Pasadena, CA) and the output was presented as a percentage of parasitized erythrocytes (PPE). Statistical analysis was performed using two-way ANOVA at a significance level of 0.05.

Given the fact that optimum neutralization activity of the polyclonal anti-CLAMP antibody was observed at 72 h post infection, percentage inhibition of merozoites was calculated at this time point. The formula: 100 − [(Test – nRBC / iRBC − nRBC) × 100]^39^ was used to determine the percentage of merozoites that were inhibited at the three antibody dilutions, and the results were compared to percentage inhibition in the presence of pre-immunization serum.

### Isolation and identification of equine erythrocyte proteins that interact with CLAMP

#### Crosslinking and co-immunoprecipitation of interacting proteins

To crosslink erythrocyte surface proteins to CLAMP, 5 ml of uninfected equine erythrocytes suspended in 10 ml of 1X PBS were incubated with 5 × 10^8^ T*. equi* merozoites for 1 h at 4 °C. The Thermo Scientific DTSSP crosslinker was added to the suspension to a final concentration of 2 mM, and further incubation was performed on ice for 2 h. Tris was then added to the solution to a final concentration of 20 mM, and incubation was performed at room temperature for 15 min to quench the reaction.

To crosslink intramembrane and intracellular erythrocyte proteins to CLAMP, 5 ml of *T. equi* infected erythrocytes at ~ 11% PPE were washed twice in 1X PBS to remove *T. equi* growth media prior to being suspended in 10 ml of 1X PBS. The Thermo Scientific DSP crosslinker was then dissolved in DMSO to a final concentration of 25 mM prior to being added to the suspended erythrocytes to a final concentration of 2 mM. Incubation was performed on ice for 2 h and tris was added to a final concentration of 20 mM to quench the reaction.

The crosslinked proteins were isolated using the Thermo Scientific Pierce Co-Immunoprecipitation kit in accordance with the manufacturer’s instructions, and agarose resins supplied by the manufacturer were used as negative controls. SDS-PAGE analysis was performed using the Invitrogen NuPAGE 4–12% Bis–Tris protein gel for detection of CLAMP’s interacting partners, and immunoblot analysis was conducted as described herein to confirm that CLAMP was isolated alongside its interacting partners.

#### Mass spectrometry analysis

The protein gel was stained overnight with the Thermo Scientific Pierce Coomassie Brilliant Blue R-250 prior to being washed twice with double distilled water at 15 min each. Protein bands on the gel were cut-out and transferred into sterile 2 ml Eppendorf tubes, and trypsin digestion was performed as previously described^40^. Nano LC–MS/MS was performed to analyze the digested peptides using the Thermo Scientific Easy-nLC 1000 Ultra-High-Pressure Liquid Chromatography connected to the Thermo Scientific Orbitrap Fusion Tribrid mass spectrometer. The Waters NanoAcquity HSS T3 column with a Thermo Scientific Trap Column was used to separate the peptides as previously described^41^. Full MS scans (MS1) were acquired from peptides eluted from the orbitrap’s electrospray source and MS2 scans were conducted on 3 s timed scans that were data-dependent and were detected in the ion trap as previously described^41^.

The Thermo Scientific Proteome Discoverer software (version 2.2) was used to search the LC–MS/MS raw data against the *Equus caballus* proteome sequences available in Uniprot (www.uniport.org) to identify equine erythrocyte proteins that interact with *T. equi* CLAMP. The static peptide and dynamic modification analyses, and a decoy database search to control for false discovery rate (FDR) were performed at previously described settings^41^.

## Supplementary Information

Supplementary Information
