# Supplementary material for: Theileria equi claudin like apicomplexan microneme protein contains neutralization-sensitive epitopes and interacts with components of the equine erythrocyte membrane skeleton
Source: Sci Rep. 2021 Apr 29;11:9301. doi: 10.1038/s41598-021-88902-4 (PMC8085155; doi:10.1038/s41598-021-88902-4)
Supplement: Supplementary file 1 — Supplementary Information [file 41598_2021_88902_MOESM1_ESM.pdf]

Theileria equi claudin like apicomplexan microneme protein contains neutralization-sensitive epitopes and interacts with components of the equine erythrocyte membrane skeleton

Cynthia K. Onzere[1]*, Lindsay M. Fry[1,2], Richard P. Bishop[1], Marta G. Silva[1], Reginaldo G. Bastos[1], Donald P. Knowles[1†] & Carlos E. Suarez[1,2†*]

*Corresponding authors: Carlos E. Suarez (email: carlos.suarez@usda.gov) and Cynthia K. Onzere (email: cynthia.onzere@wsu.edu)

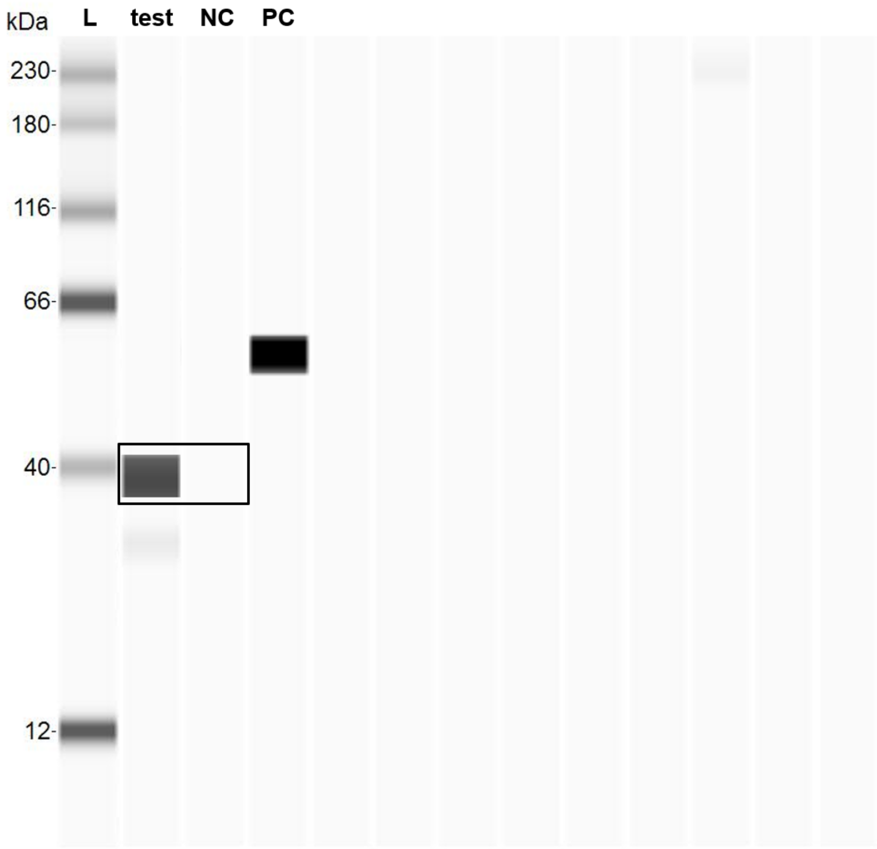

Supplementary Figure 1: A full-length immunoblot showing expression of CLAMP (within black boundary) by *T. equi* merozoites. Probing of merozoites using pre-immunization serum and *T. equi* RAP-1a specific antibody were used as negative control (NC) and positive control (PC) respectively.

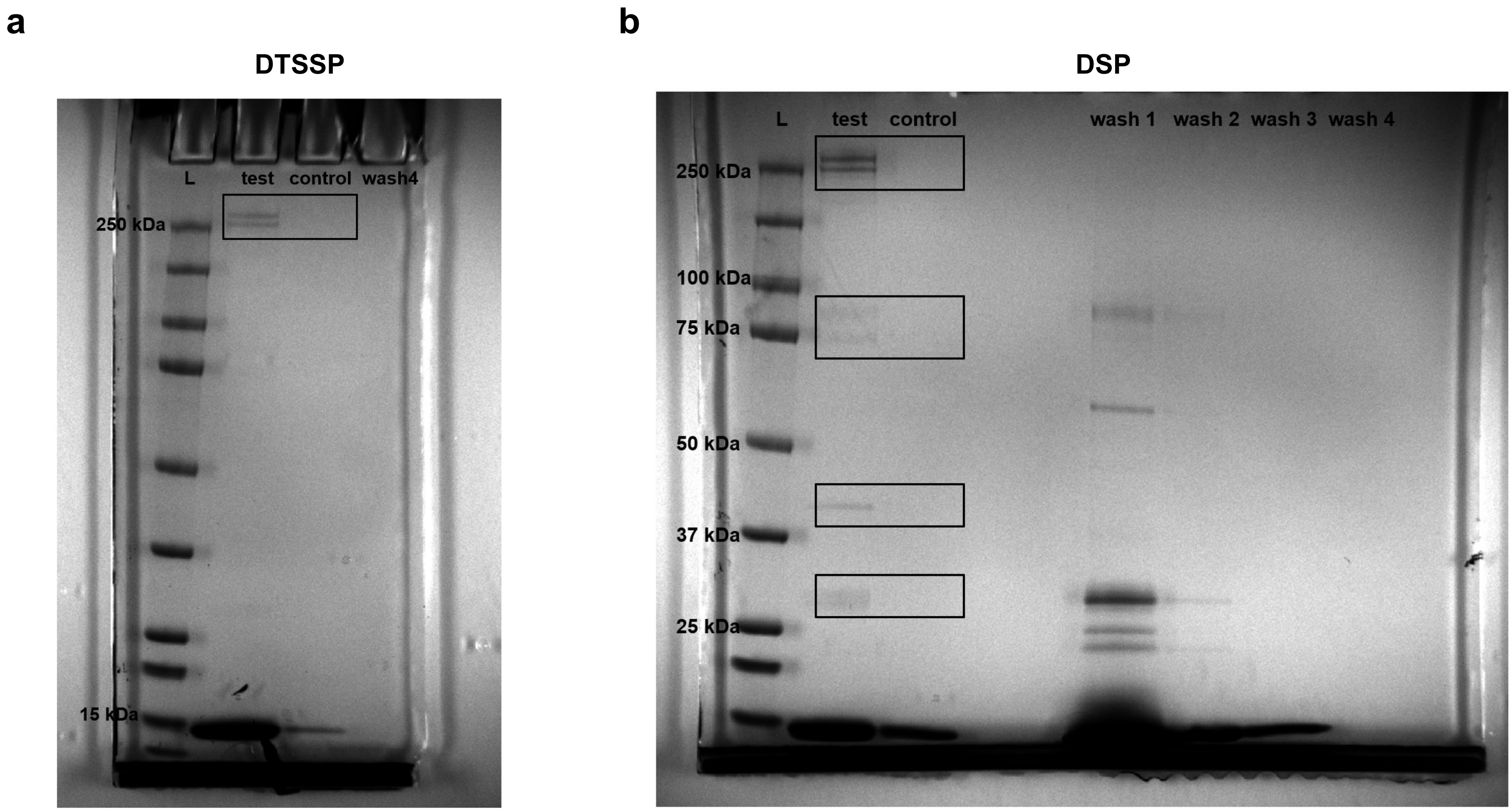

Supplementary Figure 2: SDS-PAGE gels showing equine erythrocyte proteins that interact with CLAMP within black boundaries. (a) Highlights an excised gel showing equine erythrocyte surface proteins that interact with *T. equi* CLAMP after crosslinking by the DTSSP crosslinker. (b) Represents a full-length gel showing equine intramembrane and intracellular proteins that interact with CLAMP after crosslinking by the DSP crosslinker. In both cases the washes (wash 1 to wash 4) were run alongside the eluates to verify that all unbound proteins were washed off thus confirming that only the co-immunoprecipitated proteins were eluted.

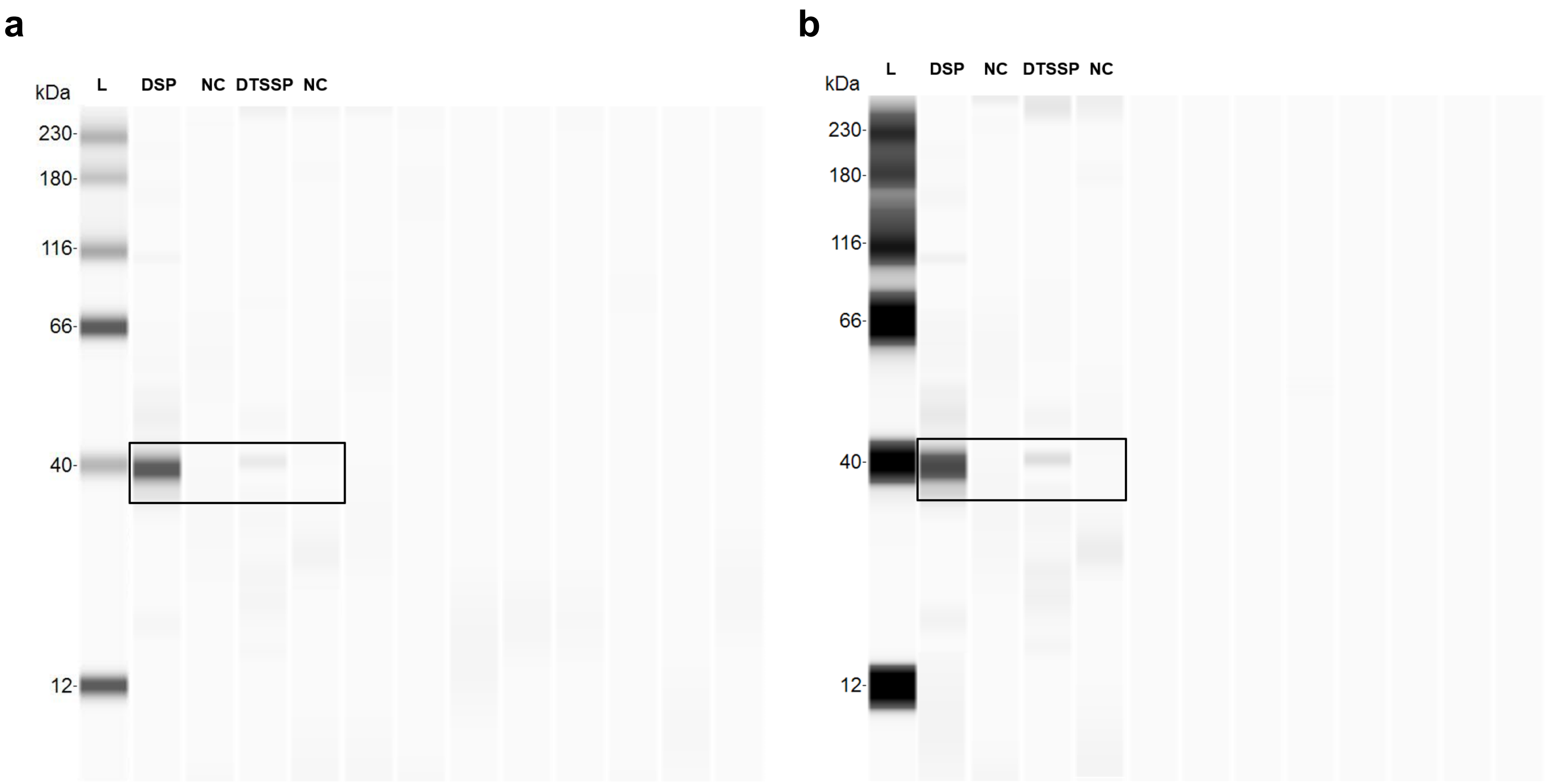

Supplementary Figure 3: Full-length immunoblot at two different contrast settings confirming elution of CLAMP alongside its interacting partners in the intramembrane and intracellular compartments (DSP) and the surface (DTSSP) of equine erythrocytes. (a) Represents the immunoblot at normal contrast levels and (b) shows the immunoblot at increased contrast levels to highlight presence of CLAMP in the DTSSP-crosslinked eluate.

Supplementary Table 1: Tandem mass spectrometry output showing equine erythrocyte proteins that interact with T. equi CLAMP

Crosslinker	Uniprot Accession Number	Protein ID	Number of Unique Peptides	Molecular Weight [kDa]	Score Sequest HT: Sequest HT	Sum PEP score
**DTSSP**	F6ZK25	Spectrin alpha, erythrocytic 1	65	280.9	360.07	140.158
	F6SIV4	Spectrin beta chain	60	268.2	339.65	135.193
**DSP**	F6ZK25	Spectrin alpha, erythrocytic 1	92	280.9	607.87	265.365
	F6SIV4	Spectrin beta chain	78	268.2	633.25	210.043
	Q2Z1P9	Anion exchange protein	17	104.2	177.92	41.91
	A0A3Q2H952	Band 4.1	15	110.4	142.76	43.26
	F6T3Y8	Actin, cytoplasmic 1	12	41.9	45.78	21.363
	A0A5F5PGV6	Tropomyosin 3	10	28.9	54.08	23.648
